# Intravitreal injections of anti-VEGF agents and antibiotic prophylaxis for endophthalmitis: A systematic review and meta-analysis

**DOI:** 10.1038/s41598-017-18412-9

**Published:** 2017-12-22

**Authors:** Manuel F. Bande, Raquel Mansilla, María P. Pata, Maribel Fernández, María José Blanco-Teijeiro, Antonio Piñeiro, Francisco Gómez-Ulla

**Affiliations:** 1 Department of Ophthalmology, University Hospital of Santiago de Compostela, Ramon Baltar S/N, 15706 Santiago de Compostela Spain; 2 Biostatech, Advice, Training and Innovation in Biostatistics, Edificio Emprendia, Campus Vida, s/n, 15782 Santiago de Compostela Spain

## Abstract

We performed a systematic review and meta-analysis to determine whether the use of local antibiotics is a beneficial prophylactic treatment for endophthalmitis in patients treated with anti-VEGF agents. We searched the MEDLINE and EMBASE databases, and the Cochrane Library over the period January 2007 to December 2016. The search terms used included “Endophthalmitis”, “Antibiotic” and “Intravitreal injection”. Studies in which the patients were treated exclusively with intravitreal injections of anti-VEGF were selected. Eight studies fit the inclusion criteria, which included a total of 276,774 injections; 109,178 (39.45%) were associated with the use of antibiotics and 114,821 (60.55%) were not associated with the use of antibiotics. Our meta-analysis indicated a significant risk for endophthalmitis that was 1.70 times greater with the use of antibiotics than that without antibiotics, with a confidence interval of 1.08 to 2.66 (p = 0.02). A meta-regression indicated that the location (operating rooms versus outpatient clinics) of injection did not have a significant effect on the incidence of endophthalmitis. The prophylactic use of antibiotics when administering anti-VEGF intravitreal injections may contribute to a greater incidence of endophthalmitis. This finding, in addition to reducing costs, would eliminate a treatment that has been shown to be unnecessary and even harmful to patients.

## Introduction

Intravitreal anti-vascular endothelial growth factor (VEGF) therapy is frequently used as one of the main treatments for many retinal pathologies, including age-related macular degeneration (exudative)^1^, diabetic macular edema^1,2^ and retinal vein occlusion^3^. The use of anti-VEGF agents has increased in recent years and now constitutes one of the most common procedures in ophthalmology. It is projected that this trend will continue for the next few years. Although the risk of injection-associated endophthalmitis is low (approximately 1 in 3000 injections)^4^, the visual morbidity of this complication is devastating^5^.

The only preventive measure with some consensus regarding its effectiveness is the application of povidone-iodine to the ocular surface prior to injection, which is the only effective prophylactic measure supported by clinical trials^6^. Other measures, such as the use of gloves, a blepharostat or masks and the setting where injection was given (operating rooms or outpatient clinics), remain controversial^7,8^. The use of antibiotics as a method of prophylaxis is part of the normal practice of intraocular procedures. However, recent studies have shown that antibiotics offer no protection against the risk of developing endophthalmitis once anti-VEGF injections have been administered, and, in some cases, the rates of endophthalmitis are actually higher in groups using antibiotics^9,10^. Although the abandonment of antibiotics before and after intravitreal administration of anti-VEGF appears to be the current trend, the use of antibiotics remains part of the normal procedure for a significant percentage of practitioners^11,12^.

Currently, no consensus exists regarding the benefits of antibiotic prophylaxis of endophthalmitis after anti-VEGF injections. To clarify this controversy, we present a meta-analysis to evaluate the incidence of endophthalmitis after treatment with anti-VEGF agents associated with 1) use of topical antibiotics and 2) the setting where the injection is performed.

## Results

An original search produced 779 possible articles. Removal of duplicates and application of selection criteria reduced the number of articles comparing two groups to eight. As shown in these publications, the proportion of cases of endophthalmitis among the total patients analyzed in each publication was very low (0.035%), with values ranging from 0.012% to 0.100%. In cases of endophthalmitis with and without antibiotic prophylaxis, staphylococcus and streptococcus were the most frequent microorganisms identified in culture-proven endophthalmitis. In six of the studies, the injections were administered in an operating room, and in the two remaining studies, the injections were administered in outpatient clinics. Table 1.

**Table 1:** Summary of studies selected for inclusion in the meta-analysis.

Study	Place	Timing (AB)	Injections	Endoph	Inject. AB	Endoph. AB	Inject. no AB	Endoph. no AB
Falavarjani *et al*.^33^	2	2	5,901	6	3,975	6	1,926	0
Cheung *et al*.^*^^34^	1	2	14,960	7	10,061	6	4,899	1
Fineman *et al*.^8^	1	2	10,164	3	7,415	2	2,749	1
Mason *et al*.^35^	1	2	5,233	1	2,617	1	2,616	0
Park *et al*.^36^	1	1	16,186	2	8,078	0	8,108	2
Falavarjani *et al*.^37^	2	2	8,037	1	2,771	1	5,266	0
Storey *et al*.^38^	1	3	147,479	52	57,654	28	89,825	24
Li *et al*.^*^^39^	1	3	68,814	15	16,607	4	52,207	11

Codes: Place: 1 = Outpatient clinics, 2 = Operating room; AB: 1 = Pre-injection, 2 = Post-injection, 3 = Pre- and post-injection, 4 = No AB. Abbreviations: Injec.: injections, Endoph.: endophthalmitis, and AB: antibiotic. *Extra information provided by the authors.

### Relationship between antibiotic therapy and endophthalmitis

To perform this meta-analysis, we selected publications that indicated both the number of anti-VEGF injections associated with the use of antibiotics as well as the number of injections not associated with the use of antibiotics. The studies included in this analysis were characterized by homogeneity regarding the type of healthcare professional that administered the injections (ophthalmologist) and the use of povidone in all cases.

**Figure 1:**
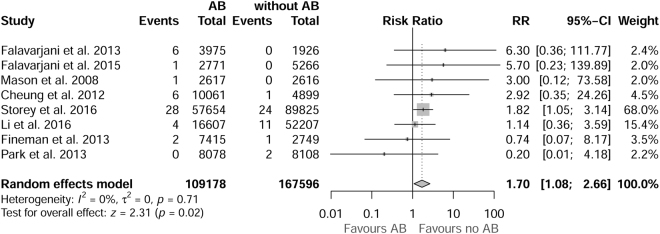
Forest plot with relative risk (RR) estimates of each study and the combined RR (represented as a rhombus), including the 95% confidence intervals and the weights assigned to each study.

Eight studies were selected, involving a total of 276,774 injections, with 109,178 (39.45%) injections associated with the use of antibiotics and 167,596 (60.55%) injections not associated with the use of antibiotics. The relative risk (RR) of endophthalmitis was 1.70 times greater with the use of antibiotics than without antibiotics, with a confidence interval of 1.08 to 2.66 (the number of cases of endophthalmitis in AB group and non-AB was 48 and 39, respectively). Therefore, the use of prophylactic antibiotics is associated with a higher incidence of endophthalmitis (z = 2.31, p = 0.02). The forest plot (Fig. 1) shows the RR and 95% confidence intervals for each of the studies and the combined RR obtained from the random effects model.

No heterogeneity was observed in the I^2^ value, which indicates a null-low heterogeneity (I^2^ = 0% (0%, 31%)), or with the test of heterogeneity (Q (degrees of freedom) = 4.52 (7), p = 0.717). Both the graphical analysis (Figure S1) and the regression test (t = 0.007, p-value = 0.994) indicated the absence of publication bias. With respect to the sensitivity analysis, after excluding the study by Story *et al*., which had a sample size much greater than the remaining sample, the RR decreased to 1.49 (0.7664, 3.3016), but heterogeneity increased substantially (I2 = 0%–61%). The number of cases of endophthalmitis in AB group and non-AB was 20 and 15 respectively, when Storey *et al*. was excluded from the analysis.

### Meta-regression analysis of the effect of the setting where injection was given

Meta-regression was performed to assess whether the setting where injection was administered (operating room versus outpatient clinics) influenced the incidence of endophthalmitis associated with antibiotic treatment. The analysis results (z = 1.165, p = 0.243) suggest that the setting where the injection was administered had no significant effect on the incidence of endophthalmitis associated with antibiotics.

Moreover, the results of the various meta-regressions showed no significant effect of any of the following variables on the incidence of endophthalmitis associated with antibiotic treatment: (a) the time at which the antibiotic was administered (pre- versus post-injection antibiotics): QM = 2; 42, p = 0.298 and (b) the causative microorganism: QM = 0; 02, p = 0.905.

## Discussion

This study presents a meta-analysis that indicates an increased risk of endophthalmitis in patients treated with intravitreal anti-VEGF agents who were also treated with prophylactic topical antibiotics. Our analysis also revealed no significant differences in the risk of developing endophthalmitis for patients who received injections in the operating rooms versus those who received injections in outpatient clinics.

We have also attempted to conduct this study in as rigorous a manner as possible and took extra precautions to control for heterogeneity across studies and publication bias to achieve maximum reliability of the results. The main assumption casting doubt on the validity of the meta-analysis is the lack of significant variations in the procedures for various valued tests. Another factor that adds value to our statistical analysis is the importance of the study by Storey *et al*. to our conclusions. Increasing the sample size is very important when studying diseases with low incidence such as endophthalmitis

Recent studies have indicated that the use of topical antibiotics could increase resistance to some antibiotics by affecting the conjunctival and nasopharyngeal flora. Moreover, increasing the proportion of resistant bacteria on the ocular surface increases the risk of developing antibiotic-resistant infections that are difficult to treat^13^. Several possibilities could explain the trend of increasing antibiotic resistance. Patients receiving anti-VEGF intravitreal injection therapy for retinal diseases often require repeated doses for long periods of time. Our data confirm that short courses of topical antibiotics affect the patterns of resistance of periocular flora. Fluoroquinolones are the most commonly used post-injection prophylactic antibiotics in patients due to their broad spectrum and high penetration. Several studies have demonstrated substantial levels of resistance to third- and fourth-generation fluoroquinolones, as well as multi-drug resistance in patients treated with topical antibiotics after multiple intravitreal injections^14,15^. On the other hand, it would be necessary to take into account the cost saving that may result from the non-instillation of antibiotic eye drops in patients undergoing this type of treatment.

Among other factors that could influence the risk of developing intraocular infection, consideration of the location of treatment administration is essential. A study based on an analysis of 12,249 injections performed by the same surgeon showed significantly lower levels of endophthalmitis when the injection was performed in the operating room instead of outpatient clinics^16^. By contrast, another study showed that the rate of endophthalmitis after an intravitreal injection was low regardless of whether the procedure was performed in outpatient clinics or in an operating room (0.035 versus 0.065%, respectively)^17^. The results of our meta-analysis support this finding.

Recently, Dutheil *et al*.^18^ presented a similar meta-analysis based on various types of intravitreal injections, including triamcinolone, dexamethasone and perfluoropropane (CF8). The goal of our work was to improve the protocol for the use of anti-VEGF drugs. The inclusion of corticosteroids in an analysis of the rates of endophthalmitis in intravitreal injections may interfere with this objective. Some studies support the idea that the risk of endophthalmitis is not equal among the possible options for intravitreal treatment. Triamcinolone presents a significant increase in the OR of 6.92 for endophthalmitis compared to anti-VEGF agents^19^. This result is partly explained by the immunosuppression that corticosteroids produce in the ocular microenvironment^20^.

However, it is necessary to consider of the following limitations in our study: methodological limitations due to a lack of access to patient data-level statistics, sensitivity bias and considering only those manuscripts published in English. All eight studies included in our analysis were retrospective reviews; they were all case-control studies rather than randomized controlled trials due to the fact that the evaluated randomized trials did not met inclusion criteria. Some of case-control studies were carried out in different periods of time, which may have carried changes in hospital prophylaxis procedures; those aspects could not be considered when evaluating the data. Furthermore, none of the studies provided differentiation of positive cultures between groups with/without antibiotic use; therefore, this variable could not be analyzed in our study. However, the data from the eight studies were sufficient to assess the duration of antibiotic treatment and the RR. Therefore, although our findings seem conclusive, these limitations suggest that the results of this study should be interpreted with caution.

We ultimately believe that the decision to use antibiotics in the prophylactic period depends on individual ophthalmologists. Ophthalmologist must analyze the evidence supporting the practice of antibiotic prophylaxis and the evidence contraindicating the use of antibiotics, as the overuse of antibiotics could possibly cause the creation and proliferation of resistant strains and increase drug costs and the likelihood of possible adverse reactions to the drugs administered.

This study included the largest meta-analysis published on this subject to date. The results of our study establish that the prophylactic use of antibiotics for intravitreal anti-VEGF injections is associated with a higher incidence of endophthalmitis. This finding could potentially eliminate an unnecessary intervention that is likely harmful to patients. Additionally, we did not observe any benefits associated with the location at which the administration of the intravitreal injection was performed, such as during a consultation at an outpatient clinic as opposed to during surgery or at another location. These two observations have implications for patient comfort, efficiency and the costs of administering these treatments.

## Materials and Methods

The Meta-analysis Of Observational Studies in Epidemiology (MOOSE)^21^ criteria along with the Preferred Reporting Items for Systematic Reviews and Meta-Analyses (PRISMA)^22^ were used for this review and meta-analysis (Table S1).

### Search methods used to identify studies

We searched MEDLINE, EMBASE and the Cochrane Database of Systematic Reviews from January 2007 (starting date of treatment injecting intravitreal anti-VEGFs) to December 2016. The terms used included “Endophthalmitis”, “Antibiotic” and “Intravitreal injection”. Different combinations of terms/descriptors were used to ensure the inclusion of a greater number of studies. For details of the search strategy, please see the supplementary materials (Table S2).

### Selection criteria for studies

During these investigations, specific predetermined selection criteria were applied to individually assess the eligibility of the studies. The inclusion criteria were as follows: (1) studies published in English, (2) studies that produced original data on the problem and (3) studies examining the incidence, prevalence or risk of post-injection endophthalmitis associated with prophylactic topical antibiotics administered before or after injections of anti-VEGF (ranibizumab, bevacizumab and aflibercept) and prophylactic administration of antibiotics (independent variables).

The exclusion criteria were as follows: (1) studies in which patients were treated with intravitreal anti-VEGF injections in combination with another therapeutic strategy (for example, photodynamic therapy) and (2) studies involving intravitreal triamcinolone injections or another type of steroid used as a main therapeutic agent or combined with other anti-VEGF agents, which is justified because the delimitation only includes a single class of agents, inhibitors of VEGF. We attempted to specifically exclude intravitreal corticosteroids among articles that met the criteria of homogeneity with the rest of the meta-analyses. This process was performed by collaboration of the authors. Table S3 details the PICOS (population, interventions, comparators, outcomes and study.

### Study selection, data collection and risk of bias assessment

**Figure 2:**
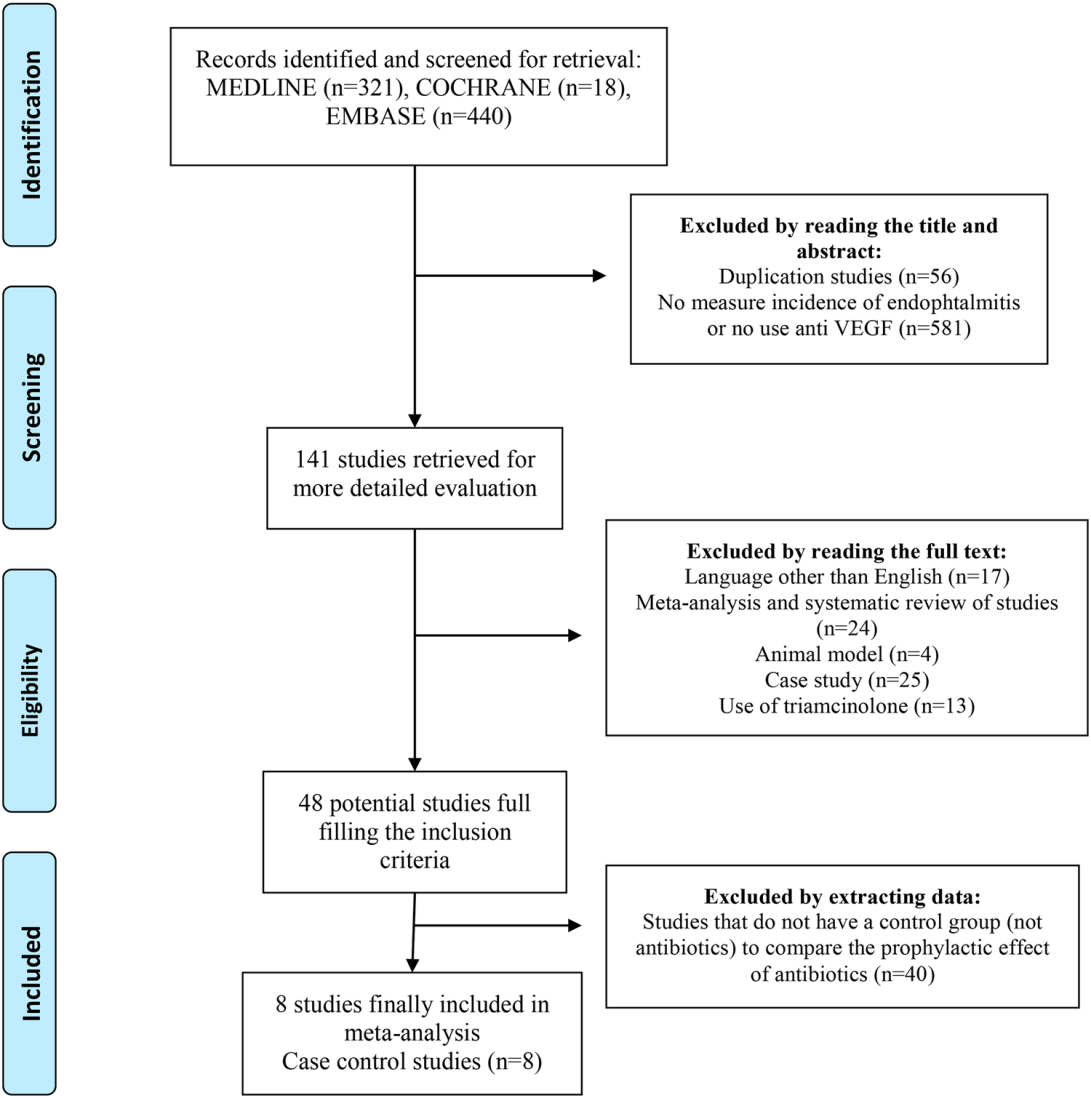
Identification and selection of studies for the meta-analysis.

One author (MB) conducted all literature searches and collated the abstracts. Two authors (MB and RM) separately reviewed the abstracts and determined the suitability of the articles for inclusion in the study based on the selection criteria. Any disagreement was resolved through discussions with a third reviewer (AP). In addition, reference lists of all publications meeting the inclusion criteria were manually searched to identify any further studies not identified via electronic searches. The search strategy is described in Fig. 2. To assess the methodological quality of the studies, we used the Strengthening the Reporting of Observational Studies in Epidemiology Statement (STROBE) checklist for observational studies^23^.

Publication bias was assessed graphically using a funnel plot and a Galbraith plot^24^ and via regression tests for funnel plot asymmetry^25^. Identification of publications that could influence the outcome of the study was conducted with the leave-one-out sensitivity analysis^26^.

For quality assessment analysis, the following items were evaluated in each of the publications: (1) Research question explicitly defined, 2) Sample size, 3) Characteristics and definition of study population, 4) Specification of inclusion and exclusion criteria, 5) Good definition and assessment (accurate and reliable methods), 6) Statistical analysis, 7) Completeness of reporting, 8) Study design, 9) External validity of results 10) conflict of interest in the conduct of the study.

These ten items were evaluated for each of the individual studies, assigning a three-level code (1: poor reporting/quality, 2: acceptable, 3: good). The quality score for each of the papers included in the analysis was between acceptable and good quality/reporting.

### Data synthesis and analysis

The primary outcome measure was the relative risk (RR) of endophthalmitis. The random effects model with restricted maximum likelihood estimation (Restricted Maximum Likelihood, REML) was used^27^. The existence of statistical heterogeneity between studies was evaluated using the I^2^ index^28,29^.

A subgroup analysis was also performed to assess the effect of certain covariates on the RR of endophthalmitis. These analyses were conducted using meta-regression^30^ with mixed model effects^27^.

All analyses were performed using the metafor^31^ library in R software (R Core Team, 2016)^32^.

### Precis

The use of antibiotics in the prophylaxis of endophthalmitis when administering intravitreal injections of anti-VEGF agents may be harmful. Our meta-regression analysis also indicated that outcomes do not vary with respect to the setting where injection was given (outpatient clinics or operating rooms) of antibiotic administration.

## Electronic supplementary material

Supplementary Info
