# Supplementary material for: Intravitreal injections of anti-VEGF agents and antibiotic prophylaxis for endophthalmitis: A systematic review and meta-analysis
Source: Sci Rep. 2017 Dec 22;7:18088. doi: 10.1038/s41598-017-18412-9 (PMC5741717; doi:10.1038/s41598-017-18412-9)
Supplement: Supplementary file 1 — Supplementary Info [file 41598_2017_18412_MOESM1_ESM.pdf]

TITLE:

Intravitreal injections of anti-VEGF agents and antibiotic prophylaxis for endophthalmitis: A systematic review and meta-analysis

AUTHORS:

Manuel F. Bande M.D.[1]
Raquel Mansilla M.D. [1]
María P. Pata PhD. [2]
Maribel Fernández M.D. PhD. [1]
María José Blanco-Teijeiro M.D. PhD. [1]
Antonio Piñeiro M.D. PhD. [1]
Francisco Gómez-Ulla M.D. PhD. [1]

Supplementary Figure 1: Plots for assessment of publication bias in the studies included in the meta-analysis.

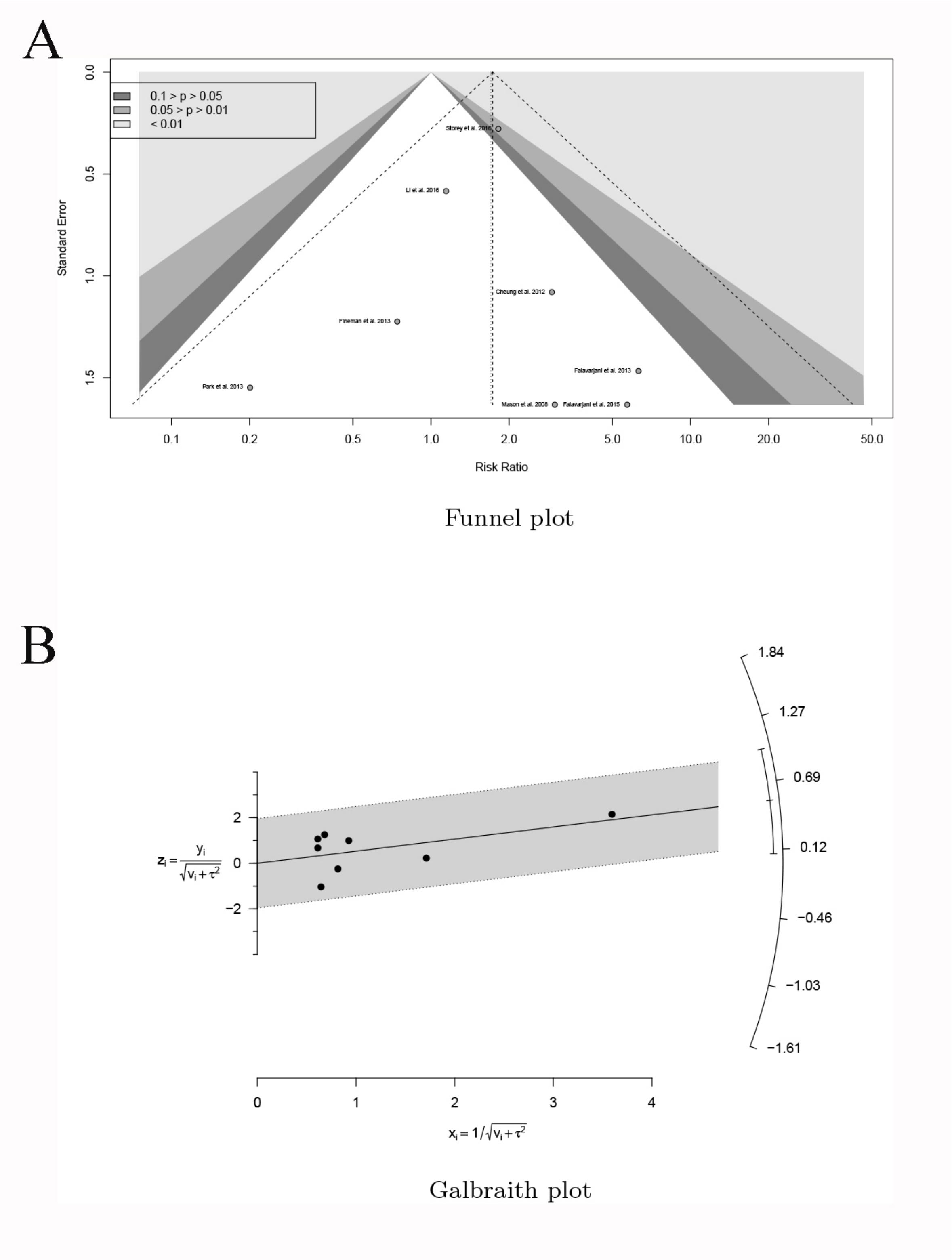

Supplementary Table 1. The PRISMA checklist.

Section/topic		Checklist item	Reported on page #
**TITLE**			
Title	1	Identify the report as a systematic review, meta-analysis, or both.	1
**ABSTRACT**			
Structured summary	2	Provide a structured summary including, as applicable: background; objectives; data sources; study eligibility criteria, participants, and interventions; study appraisal and synthesis methods; results; limitations; conclusions and implications of key findings; systematic review registration number.	2
**INTRODUCTION**			
Rationale	3	Describe the rationale for the review in the context of what is already known.	3
Objectives	4	Provide an explicit statement of questions being addressed with reference to participants, interventions, comparisons, outcomes, and study design (PICOS).	6,7, Appendix
**METHODS**			
Protocol and registration	5	Indicate if a review protocol exists, if and where it can be accessed (e.g., Web address), and, if available, provide registration information including registration number.	6
Eligibility criteria	6	Specify study characteristics (e.g., PICOS, length of follow-up) and report characteristics (e.g., years considered, language, publication status) used as criteria for eligibility, giving rationale.	6
Information sources	7	Describe all information sources (e.g., databases with dates of coverage, contact with study authors to identify additional studies) in the search and date last searched.	6
Search	8	Present full electronic search strategy for at least one database, including any limits used, such that it could be repeated.	6, Appendix
Study selection	9	State the process for selecting studies (i.e., screening, eligibility, included in systematic review, and, if applicable, included in the meta-analysis).	6
Data collection process	10	Describe method of data extraction from reports (e.g., piloted forms, independently, in duplicate) and any processes for obtaining and confirming data from investigators.	7
Data items	11	List and define all variables for which data were sought (e.g., PICOS, funding sources) and any assumptions and simplifications made.	6, Appendix
Risk of bias in individual studies	12	Describe methods used for assessing risk of bias of individual studies (including specification of whether this was done at the study or outcome level), and how this information is to be used in any data synthesis.	7
Summary measures	13	State the principal summary measures (e.g., risk ratio, difference in means).	7
Synthesis of results	14	Describe the methods of handling data and combining results of studies, if done, including measures of consistency (e.g., I^2) for each meta-analysis.	7

PRISMA 2009 Checklist

Section/topic		Checklist item	Reported on page #
Risk of bias across studies	15	Specify any assessment of risk of bias that may affect the cumulative evidence (e.g., publication bias, selective reporting within studies).	7-Appendix
Additional analyses	16	Describe methods of additional analyses (e.g., sensitivity or subgroup analyses, meta-regression), if done, indicating which were pre-specified.	2,7
**RESULTS**			
Study selection	17	Give numbers of studies screened, assessed for eligibility, and included in the review, with reasons for exclusions at each stage, ideally with a flow diagram.	7
Study characteristics	18	For each study, present characteristics for which data were extracted (e.g., study size, PICOS, follow-up period) and provide the citations.	8
Risk of bias within studies	19	Present data on risk of bias of each study and, if available, any outcome level assessment (see item 12).	7
Results of individual studies	20	For all outcomes considered (benefits or harms), present, for each study: (a) simple summary data for each intervention group (b) effect estimates and confidence intervals, ideally with a forest plot.	8-Appendix
Synthesis of results	21	Present results of each meta-analysis done, including confidence intervals and measures of consistency.	2,4
Risk of bias across studies	22	Present results of any assessment of risk of bias across studies (see Item 15).	7-Appendix
Additional analysis	23	Give results of additional analyses, if done (e.g., sensitivity or subgroup analyses, meta-regression [see Item 16]).	4-Appendix
**DISCUSSION**			
Summary of evidence	24	Summarize the main findings including the strength of evidence for each main outcome; consider their relevance to key groups (e.g., healthcare providers, users, and policy makers).	2,4-6
Limitations	25	Discuss limitations at study and outcome level (e.g., risk of bias), and at review-level (e.g., incomplete retrieval of identified research, reporting bias).	5
Conclusions	26	Provide a general interpretation of the results in the context of other evidence, and implications for future research.	6
**FUNDING**			
Funding	27	Describe sources of funding for the systematic review and other support (e.g., supply of data); role of funders for the systematic review.	8

From: Moher D, Liberati A, Tetzlaff J, Altman DG, The PRISMA Group (2009). Preferred Reporting Items for Systematic Reviews and Meta-Analyses: The PRISMA Statement. PLoS Med 6(6): e1000097. doi:10.1371/journal.pmed1000097

For more information, visit: **www.prisma-statement.org**.

Supplementary Table 2. Search strategy.

PubMed: 328 records (January 2007 to December 2016)

(("endophthalmitis"[MeSH Terms] OR "endophthalmitis"[All Fields]) AND ("anti-bacterial agents"[Pharmacological Action] OR "anti-bacterial agents"[MeSH Terms] OR ("anti-bacterial"[All Fields] AND "agents"[All Fields]) OR "anti-bacterial agents"[All Fields] OR "antibiotic"[All Fields]) AND ("intravitreal injections"[MeSH Terms] OR ("intravitreal"[All Fields] AND "injections"[All Fields]) OR "intravitreal injections"[All Fields] OR ("intravitreal"[All Fields] AND "injection"[All Fields]) OR "intravitreal injection"[All Fields])) AND ("2007/01/01"[PDAT] : "2017/12/01"[PDAT])

EMBASE: 440 records (January 2007 to December 2016)

#4 #1 AND #2 AND #3

#3 'antibiotic'/exp

#2 ' intravitreal injections'/exp

#1 ' endophthalmitis'/exp

Cochrane Library: 18 records (January 2007 to December 2016)

#1 MeSH descriptor: [Endophthalmitis] explode all trees

#2 MeSH descriptor: [Anti-Infective Agents] explode all trees

#3 MeSH descriptor: [intravitreal injections] explode all trees

#4 #1 and #2 and #3

Supplementary Table 3. PICOS criteria for inclusion and exclusion of studies

Parameter	Inclusion criteria	Exclusion criteria
**Patients**	Adults >18 years<br>Patients were treated with intravitreal anti-VEGF injections (ranibizumab, bevacizumab and aflibercept)	Patients under 18 years of age<br>Patients treated with intravitreal anti-VEGF injections in combination with another therapeutic strategy
**Intervention**	Prophylactic administration of antibiotics in patients were treated with intravitreal anti-VEGF injections	
**Comparator**	No prophylactic administration of antibiotics in patients were treated with intravitreal anti-VEGF injections	
**Outcomes**	Incidence, prevalence or risk of post-injection endophthalmitis associated with prophylactic topical antibiotics administered before or after injections of anti-VEGF (ranibizumab, bevacizumab and aflibercept) and prophylactic administration of antibiotics (independent variables).	Studies without defined clinical outcomes
**Study Design**	Randomized controlled trials<br>Non-randomized controlled trials<br>Retrospective, prospective, or concurrent cohort studies<br>Cross sectional studies	Case reports<br>Editorials & opinion pieces
